# Inward Leakage in Tight-Fitting PAPRs

**DOI:** 10.1155/2011/473143

**Published:** 2011-05-12

**Authors:** Frank C. Koh, Arthur T. Johnson, Timothy E. Rehak

**Affiliations:** ^1^Fischell Department of Bioengineering, University of Maryland, College Park, MD 20742, USA; ^2^National Personal Protection Technology Laboratory, National Institute for Occupational Safety and Health, Pittsburgh, PA 15236, USA

## Abstract

A combination of local flow measurement techniques and fog flow visualization was used to determine the inward leakage for two tight-fitting powered air-purifying respirators (PAPRs), the 3M Breathe-Easy PAPR and the SE 400 breathing demand PAPR. The PAPRs were mounted on a breathing machine head form, and flows were measured from the blower and into the breathing machine. Both respirators leaked a little at the beginning of inhalation, probably through their exhalation valves. In both cases, the leakage was not enough for fog to appear at the mouth of the head form.

## 1. Introduction

Tight-fitting powered air-purifying respirators (PAPRs) can be used in situations where contaminated air must be filtered, with the additional work of drawing air through the filter supplied by a battery-powered blower rather than the wearer's respiratory muscles. Tight-fitting PAPRs form a tight seal with the face, which should minimize exposure to contaminated air. Maintenance of a positive pressure within the facepiece portends that any face seal leakage that does occur should flow outward rather than inward. Performance of work while wearing PAPRs in the heat is influenced also by the cooling flow of air across the face [1].

Despite these advantages, some doubts remain. Is the protection afforded by tight-fitting PAPR wear as good as it would seem? Is there opportunity for contamination to enter the facepiece and be inhaled by the wearer? Is inward leakage, if it exists, likely to lead to inhaled contaminant?

In this study, we have been motivated to explore these issues. Of importance to the wearer is contaminant-laden air that reaches the mouth [2]. This study was conducted to determine if (1) there is possible leakage of ambient air into the PAPR facepieces and (2) whether contaminant leakage actually reaches the mouth where it can be inhaled. The second objective has been considered to be the more important of the two because it relates directly to the safety and health of the wearer. The first objective can be achieved by measuring blower flows and comparing them to inhaled flows. The second objective could be accomplished by visual detection inside the PAPR facepiece, especially at the mouth. The major hurdle to overcome is the fact that tight-fitting PAPRs almost always include visually opaque facepieces, unlike loose-fitting PAPRs. Thus, leakage flow pathways were not able to be visualized in this study.

## 2. Methods

Two commercially available tight-fitting PAPRs (SE 400; SEA, Meadowlands, PA; 3M Breathe Easy; St. Paul, MN) were tested on a breathing machine head form (Krug Life Sciences, Houston, TX). The SE 400 PAPR is a breathing-demand device with a blower that adjusts to the breathing flow rates of the wearer. The 3M Breathe-Easy PAPR has a blower that is supposed to supply a constant 114 L/min flow rate to the facepiece (this figure was obtained from the manufacturer's literature). The SE 400 blower was operated with a fully charged battery; the 3M Breathe-Easy blower had accessible electrical connections and was attached to a dc power supply at 4.8 volts in order to assure a constant rate of flow. For each of these tests, the Krug breathing machine was operated at 40 breaths/min and with a tidal volume of about 2.25 L.

**Figure 1:**
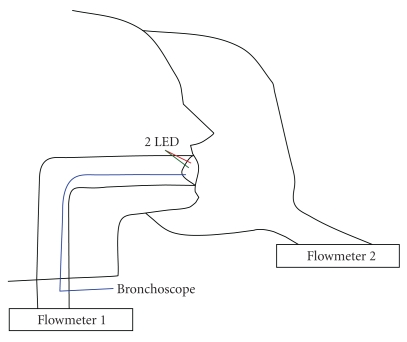
Schematic diagram of the experimental arrangement with flow meters, light-emitting diodes, PAPR, head form, and bronchoscope.

Both PAPRs were installed, in turn, on the head form and the head straps adjusted for tight, uniform fits (Figure 1). One set of measurements was made on each PAPR in this unmodified condition. However, the surface of the head form is hard and smooth, certainly not like the flesh of the face. Because of this, any leakage that would occur around the periphery of the head form would not necessarily be indicative of leakage that would occur around the periphery of a human wearer. Therefore, two additional sets of measurements were made with ample modeling clay applied around the facepiece periphery to obstruct any inadvertent leaks formed at the face seal. One of these compared blower and breathing machine flow rates, and the other focused on the exhalation valves as the most likely sources of external leakage.

Two MedGraphics (St. Paul, MN) no. 5038773 pitot-tube flowmeters were used, one measuring blower flow to the facepiece and another measuring flow into the breathing machine. The difference between the two measured flow rates would indicate leakage somewhere in the system. When inhaled flow rate exceeded blower flow rate, the leakage was inward. This difference, when integrated over the inhalation time, gave the total volume of air leaked from the outside into the facepiece during a single inhalation. The flow meter measuring blower flow on the SE 400 was attached to the inlets of the filters with a custom-made adapter; the flow meter for the 3M Breathe-Easy blower was inserted in the hose between the filter and the facepiece.

Careful calibration of the flow meters was necessary because the difference in their readings was so important. Each flow meter was attached to a Validyne (Northridge, CA) DP-15 differential pressure transducer and a Validyne CD-12 demodulator transducer indicator. The transducer indicators produced an electrical voltage related to the transducer pressure. These were calibrated first with an inclined manometer; a T-connector was used to apply the same pressure simultaneously to both pressure transducers. Once the pressure transducers were calibrated and connected to the flow meters, both flow meters were calibrated with the same flow from the breathing machine. They were arranged in series with three 3.8 cm (1.5 in) diameter, 38.1 cm (15 in) long pipes to stabilize the flow velocity profile upstream from the flow meters, between the flow meters, and downstream from the flow meters. A Fleisch no. 3 (Phipps and Bird; Richmond, VA) pneumotach inside the breathing machine served as the flow reference. Regression analysis was performed with the aid of EXCEL software (Microsoft; Redman, WA).

The entire breathing machine and PAPR were located inside a phone booth-sized box of dimensions 137 cm (54 in) by 76 cm (30 in) by 180 cm (71 in). The box was constructed of a wood frame with plywood walls. The upper part of the box was made from Lexan transparent plastic material in order to see what was going on inside.

Glycerol fog was formed with a Fogstorm 1200 HD (Los Angeles, CA) generator that can generate 200 m^3^ fog per minute. This fog was introduced into the test chamber through a hose inserted through the side of the chamber. Fog permeated the entire atmosphere inside the chamber. Although no measurements were made to characterize this fog, theatrical fog usually has a concentration less than 10 mg/m^3^ with droplets 20–30 *μ*m in size [3]. 

Of primary interest was the point at which fog reached the mouth. Because both PAPRs had opaque facepieces, a bronchoscope (Pentax model B011471; Asabi Optical, Tokyo, Japan) was placed inside the tube leading to the mouth of the head form, with its tip at the mouth and facing the cavity between the PAPR facepiece and the head form. The place where the bronchoscope penetrated the head form breathing tube was sealed around the bronchoscope with silicone sealant.

The bronchoscope consisted of a bundle of optical fibers that conducted light toward the tip and an image back from the tip. The image was converted into video form by a small television camera, and the resulting image was displayed on a television monitor. Evidence of the presence of fog could be detected from the monitor.

To assure that the image produced by the bronchoscope was sufficiently sensitive to be able to detect the fog when it was present, fog was deliberately introduced at the mouth of the head form. When fog was known to be present, it was visually detectable; when no fog was present, there was no indication of fog. Although there was no direct measure of the concentration of fog as a surrogate contaminant, it could be assumed that the absence of visually detectible fog corresponded with negligible hazard.

Because it was difficult to adapt the bronchoscope television image to a form that permitted frame-by-frame analysis, a Sony (Tokyo, Japan) DCR-HC90 video camera with 3 megapixel resolution was used. An S-video jack on the bronchoscope monitor was plugged into the Sony camera, and the signal was recorded digitally at 40 frames/sec.

Also included in the bronchoscope image were two light-emitting diodes (LEDs) located at the mouth of the head form. A green LED driven by a 10 Hz square-wave electrical signal was used to help synchronize recorded images. A red LED was connected to a comparator circuit that detected inhalation. In this way, image timing and breathing phase were able to be positively identified. No accessory lighting was necessary to illuminate the dark inside of the facepiece. 

After it became apparent from the flow rate differential that there was a source of leakage into the facepiece, clay was used to seal around the facepiece periphery and the test was repeated. When leakage was still detected, attention turned to the exhalation valves. With modeling clay still in place, the test was again repeated, but the video camera was moved from the bronchoscope monitor and aimed at the exhalation valve, so that it could detect valve actions. Duplicate green and red LEDs outside the facepiece maintained the ability to keep track of time and phase of the breathing cycle. The exhalation valve cover from the 3M Breathe Easy was removed for better viewing. The valve cover from the SE 400 did not have to be removed and was kept in place. 

## 3. Results

**Figure 2:**
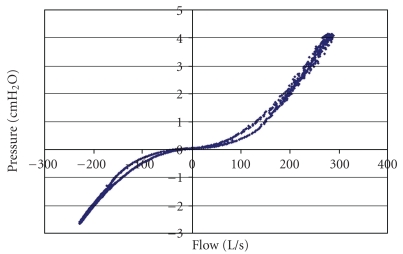
Pressure-flow relation for one of the Medgraphics flow meters used in this study. There is a small amount of hysteresis present in the data, but the hysteresis was the same for both flow meters.

**Figure 3:**
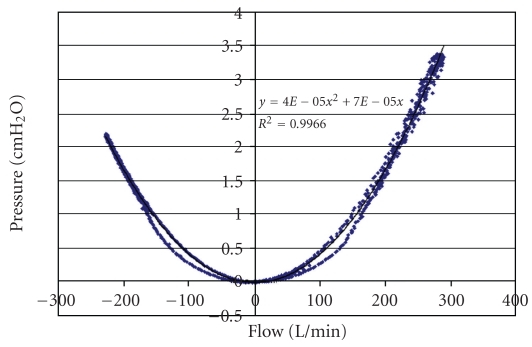
Pressure-flow relation for the flow meter with negative pressures inverted. The result is a parabola that can be used to describe calibration results mathematically. Hysteresis was present in these data, but a parabola was fitted to only one set of data. Both flow meters exhibited the same amount of hysteresis and so gave the same relative flow reading.

**Figure 4:**
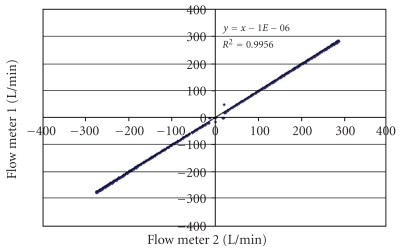
Plot of readings from the two flow meters. With an intercept of zero and a slope of one, it can be seen that both flow meters gave nearly identical results. This is important when differences between the two measured flow rates were calculated. Hysteresis data points are included in the line.

Flow meter calibration required extra care. In Figure 2 is shown the relation between pressure and flow for one of the flow meters. A Pitot-tube flow meter should theoretically relate pressure to the square of the flow. When negative calibration pressures were inverted, a complete parabola resulted (Figure 3). A quadratic regression equation forced through the origin gave a very high correlation coefficient. In order to check that both flow meters had identical characteristics, the two flow meters were connected in series to the breathing machine. The ideal regression line relating the two flow meters would be *y* = *x*, a line with slope of 1.0 and zero intercept of 0.0. From Figure 4, it can be seen that readings from both flow meters were nearly perfectly correlated.

**Figure 5:**
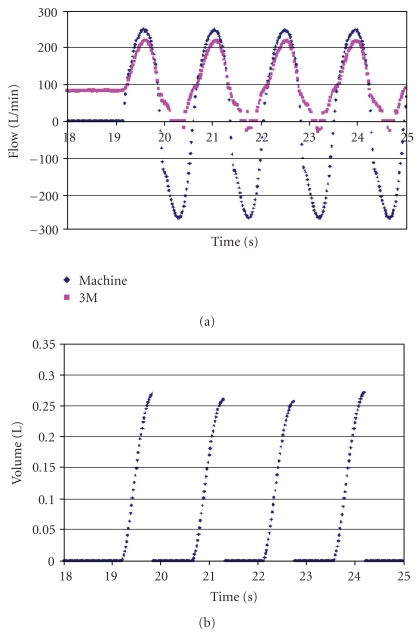
Flow rates (a) and leakage volumes (b) are plotted with time for the 3M Breathe-Easy PAPR. Leakage volumes were accumulated during inhalation when blower flows were less than breathing machine flows. No clay was used to seal the periphery.

In Figure 5 are shown the results from the 3M Breathe Easy without clay sealing the facepiece to the head form. On the left, the breathing machine was off (no flow), and the blower supplied a constant flow of nearly 100 L/min. At 19 seconds, the breathing machine was turned on, and both flows increased. Breathing machine flow can be seen to be greater than blower flow during inhalation. The difference represents leakage. This difference was integrated to obtain leakage volume during the time that blower flow was less than breathing machine flow, and the results are shown in the lower diagram of Figure 5. This shows that nearly 0.26 L of outside air leaked into the facepiece during each inhalation. This air was presumably swept from the facial volume during exhalation. 

Also to be noted here is that blower flow rate was not constant. It varied during the breathing cycle, becoming about 230 L/min during inhalation peaks and falling to nearly zero during exhalation. The power required to move the extra air through the filters must necessarily be coming from the breathing machine.

There are a few blower negative flow values during exhalation. This means that respired air is being pushed backwards through the filters. At least part of the hose between the blower and the facepiece is also being filled with respired air. This PAPR has no check valve in the blower circuit to prevent backwards flow.

**Figure 6:**
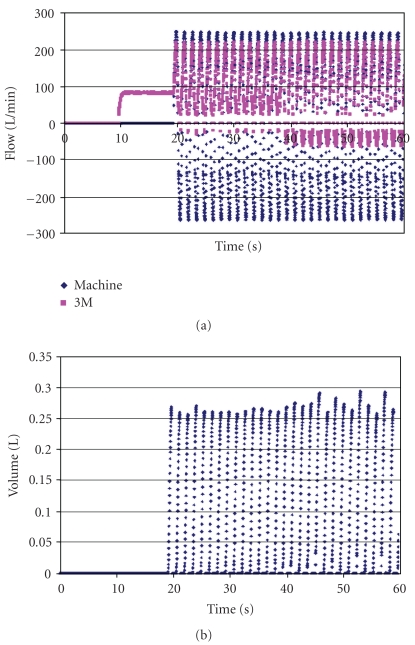
Flow rates (a) and leakage volumes (b) for the 3M Breathe-Easy PAPR shown in the previous figure. The time scale has been compressed to show effects of blower and breathing machine activity. The blower was turned on at the 10-second mark, the breathing machine was turned on at 20 seconds, and the blower was turned off at 40 seconds. The negative blower flow before and after the blower was turned off demonstrates that air was being forced backward through the blower and filter. No clay was used to seal the periphery.

In Figure 6 are shown the same two flow measurements (a) and the same volumes (b) as in the previous Figure, except that the time scale has been compressed. Both the blower and the breathing machine were off until 10 sec, when the blower was turned on. At 20 sec, the breathing machine was turned on. At about 40 sec, the blower was turned off. The same leakage volume seemed to occur whether the blower was on or off. There was more backflow of air through the filter when the blower was off.

**Figure 7:**
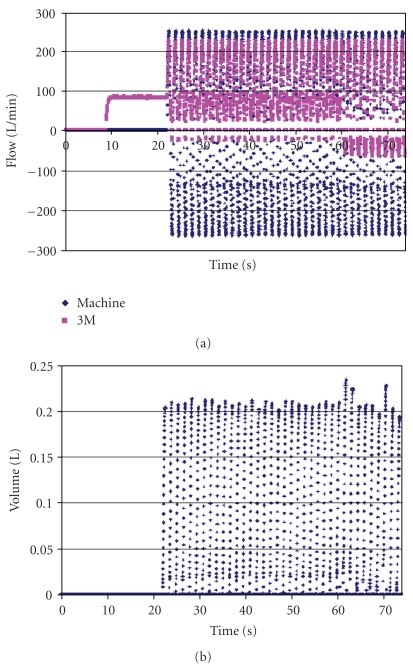
Results for the 3M Breathe-Easy PAPR when clay was carefully used to seal the periphery. The blower was turned on at about 10 seconds, the breathing machine was started at about 20 seconds, and the blower was turned off at about 60 seconds. Blower flow became negative, even while operating. Leakage volume decreased compared to the previous figure when no clay was used.

When clay was carefully used around the mask periphery, the leakage volume decreased to about 0.21 L to 0.24 L from 0.26 to 0.29 L for each inhalation (Figure 7). Again, the leakage volume did not depend upon blower activity. The bronchoscope showed no evidence of fog reaching the mouth.

**Figure 8:**
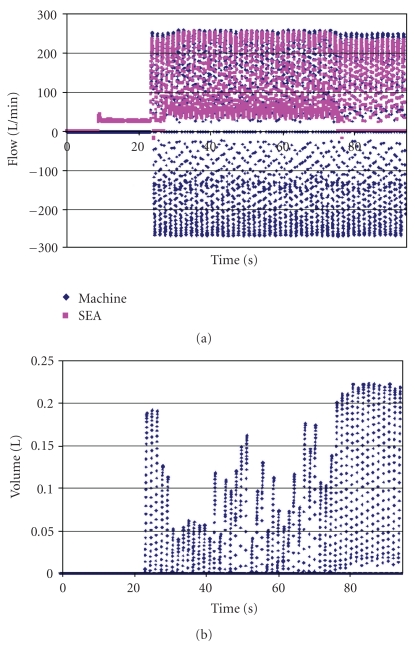
Flow rates (a) and leakage volumes (b) for the SE 400 PAPR with clay sealing the periphery. The blower was turned on just before the 10-second mark, the breathing machine was started after 20 seconds, and the blower was turned off at about 75 seconds. Except for a couple of short instances, blower flow never became negative. The extra positive blower flow when the blower was operating was used to maintain positive pressure inside the respirator. Even with the blower turned off, blower flow was not measured as less than zero.

Results for the SE 400 with clay around the face seal are shown in Figure 8. The blower was turned on at 10 seconds and maintained a steady flow rate of about 25 L/min until the breathing machine was turned on at about 25 seconds. This PAPR had an active blower control that was adjusted to breathing demand. For the first few breaths after the breathing machine was turned on, leakage volume was about 0.19 L. It subsequently decreased and varied throughout the range of 0.04 to 0.18 L. The leakage volume value seemed to depend on whether the blower was actively increasing or decreasing speed, but the exact dependence was not determined. When the blower was turned off at about 75 seconds, leakage volume increased to 0.22 L. Again, the bronchoscope detected no fog at the mouth.

Leakage volumes from both PAPRs with clay sealing their peripheries seemed to indicate that inward flow was coming through the exhalation valves. Indeed, an inspection of the videos taken of both exhalation valves showed that neither valve closed instantaneously when inhalation began. The exhalation valve on the SE 400 is larger than the corresponding valve on the 3M Breathe Easy. The SE 400 valve tended to flutter, opening about three times after inhalation began. Leakage volume for the SE 400 could not be determined because it was too difficult to see exactly when the valve was opened or completely closed. The 3M valve did not flutter but stayed open long enough to let pass about 0.01 L of air before it apparently closed (the range was measured at 0.00 to 0.03 L). This open valve leakage volume was determined as the volume of air that was measured by the two flow meters during the time that the valve was visually observed to be open. It was only a small portion of the total leakage volume measured during each inhalation. The remainder of the leakage may have come while the valve was closed or from other undetermined pathways.

## 4. Discussion

These results demonstrated that these tight-fitting PAPRs do not exclude all contaminated air from the facepiece. However, the amounts leaked are extremely small. Of particular importance to our interest is that there was no contaminant that appeared to reach the mouth of the head form. Leakage amounts in respirators are important, but the health and safety of the wearer are of primary importance. Thus, no matter what amount of leakage is present, and what pathway that it takes if it occurs, if the contaminant has no affect on the wearer, then the wearer is protected.

It was not possible for us to determine the actual pathways of the fog from the points of leakage to the mouth because of the opaque facepieces. Perhaps this could be done with miniature cameras inside the facepieces, but these were not available to us. The reason that the pathway is important is that a long enough pathway would dictate that leaked contaminants take long enough to reach the mouth that the inhalation phase of breathing would cease and contamination would be swept out of the breathing zone. Respirators designed to lengthen this pathway would presumably be more able to accommodate leakages and still protect the wearer. Knowing where leakages are likely to occur is important for this type of design. 

If the only means to detect leakage in this study was the difference between blower flow rate and inhaled flow rate, then the conclusion might have been that the wearer of either of these devices could be at risk for breathing contaminant leaking from the outside. By visualizing fog that either did or did not reach the mouth, that ambiguity was eliminated. Although a very small amount of leakage did occur, it would have had no affect on the wearer. 

Confirmation of these conclusions is based upon the detection of fog in proximity to the mouth. Tests to determine the sensitivity of visual detection of fog have shown that the methods used here are sufficient to detect the presence of fog when it should be found, and that fog was not detected when it was not present. The visual demarcation at the edge of the fog was sufficiently sharp to see the fog. It can be concluded that no intake of contaminants was measured using this technique.

There are few alternatives to measuring contaminant concentrations at the mouth. Inhalation times are very short, ranging from about 2.5 seconds at rest to 0.25 seconds during extreme exertion. Inhalation for this experiment lasted for 1.0 second. Standard particle counters require longer sampling times than this. Exotic gases may be used as contaminant surrogates, and these could be detected, but, again, required sampling times are too long. The two methods used here, differential flow rates and visual detection, can provide real-time data. 

There were no methods available to us to determine the minimum concentration of fog that could be detected, and, therefore, it cannot be said definitively what concentration level of fog reached the mouth. Thus, conclusions about the leakage hazard based upon the fog as a surrogate contaminant cannot be made for sure. Even if the fog concentration could have been determined, another atmospheric contaminant of the form of a vapor, gas, or aerosol with different properties could be transported differently within the facepiece. Hence, the fog gives an indication that contaminant leakage would not reach the mouth and be inhaled, but further studies would be necessary to determine if representative contaminants have the same behavior as the fog. Such studies would be valuable, given that contamination inside the facepiece could be tolerated as long as hazardous doses of contaminant were not inhaled. 

Because the flow pathways for the fog could not be followed from the point of entry through the space in front of the face, it was not possible to say definitively where the leakage came from. The SE 400 exhalation valve fluttered whenever the breathing machine flow was higher than its blower flow. When the valve actually closed could not be determined exactly, so the leakage volume due to valve action could not be estimated. It was easier to determine when the 3M Breathe-Easy exhalation valve closed or did not close. In both cases, leakage probably came through the exhalation valves even when apparently closed, because there just was no other obvious entry point for contaminant leakage. 

These results agree with those published years ago by Burgess and Anderson [4], who demonstrated that exhalation valve leakage was of minor consequence. They also showed that exhalation valve protective covers decrease leakage even further. Amounts of leakage in the present study were determined with protective covers in place, with the valve cover removed for the Breathe Easy only to view the valve action during exhalation. 

The breathing demand SE 400 definitely had lower leakage volume than the 3M Breathe Easy. This volume changed as the blower adjusted to the demand. Leakage in the SE 400 was often accompanied by short bursts of its negative-pressure alarm.

The dead volume of a respirator is the volume inside the facepiece that could accumulate exhaled carbon dioxide. Dead volumes of these masks were not measured but should fall in the range of about 0.97 L, which is the measured interior volume of the similar 3M FRM40 facepiece. Measured dead volume of the FRM40 nose cup is about 0.09 L. These were measured as the volumes of water filling the space between the facepiece and the head form.

What this tells us is that the leakage volumes for both respirators tested in this experiment were much less than the dead volumes of the facepieces. Contaminant leakages can occur in respirators, but if contaminants are not inhaled, then they have no health consequence. It would therefore be expected that contaminated air would not reach the mouth and be breathed by the wearer. This is confirmed by the lack of fog appearing at the mouth.

However, one might make the case that it is the volume inside the nose cup that matters. This is because the exhalation valve is located directly in front of the nose cup. Leakage coming through an imperfectly closed exhalation valve could possibly follow a direct route to the mouth. However, no fog was observed at the mouth, so leakage apparently did not follow a direct route to the mouth. An indirect flow pathway, one that lengthens the distance from the point where leakage occurs to the mouth, is an effective protective strategy. 

We also found interesting that the blower on the 3M device did not deliver a constant flow rate but instead delivered an amount dependent upon the inhaled flow rate. The extra energy needed to draw extra air across the filter, blower, and tubing resistance would have to have come from the breathing machine. If a human had to supply the same amount of energy, breathing through this respirator could become very tiring. An analysis of this situation appears in Johnson et al. [5].

The fact that air could flow backwards, even by a small amount, in the tube that supplied filtered air to the facepiece means that the effective dead volume of the device would be increased because some of the exhaled carbon dioxide could be rebreathed. The amount, as measured herein, was small, and so not likely of consequence for the average wearer. Should there be less blower flow, perhaps from lower battery voltage, then the amount of rebreathed air could become more significant. It is not likely that exhaled breath moisture could reach the filter, but, if it could, filter life could be adversely affected. A simple check valve in the supply circuit could solve the problem.

Figures 5–7 have been included in this paper despite the fact that leakages occurring at the face seal of the head form are not likely to resemble leakages that could occur at the face seal of a human. These figures have been meant to illustrate experimental techniques used and to show that face seal leakages do indeed occur on the head form. More valid to the meaning of this study are the figures of data obtained with clay sealing the peripheries of both devices. The clay was applied generously enough that no leakage could have occurred where it was used.

The steady blower flow of somewhat less than 100 L/min does not reach the NIOSH certification requirement of 115 L/min. The blower battery in this test was replaced by a power supply that maintained the fully charged battery voltage throughout the whole procedure, so the lower than expected blower flow could not have been the result of low voltage to the blower motor. Blower flow rate may have been low because of the resistance of the flow circuit through the exhalation valve when inhalation was not happening. It can be seen from the figures that blower flow rate increases significantly once inhalation starts and would possibly be high enough to pass certification testing.

If it could be maintained, a constant flow from the blower has the advantage of simplicity, with only a slight filter capacity penalty for filtering more air than is needed during less-demanding circumstances. The tidal volumes used in this testing were extremely high, as indicated previously, so smaller tidal volumes would not be as difficult for the blower to match. Even so, the respirator was able to supply the required rate of air flow. In addition, if significant amounts of contaminants were to leak into the facepiece, then additional blower flow would help to purge them from inside the respirator. 

There are two ways in which the breathing machine used in this experiment differed from a human. The first is that the breathing machine acted as an ideal flow source: its flow rate was largely independent of the resistance of the device attached to it, quite like a positive-displacement pump. The breathing machine developed whatever pressure it needed to draw the required flow rate, and this pressure was limited only by the mechanical strength of the machine.

A human respiratory system is different; first, the maximum pressure it can develop is very low in comparison to the mechanical strength of steel parts; second, this pressure varies as lung volume changes [6]; third, the respiratory system has resistance and compliance elements that make it far from an ideal flow source. Consequently, one would expect a human to adjust his flow rate to the limits of the device.

The second difference between the breathing machine and a human involves the tidal volume used. A tidal volume of 2.25 L is large for a human of medium size and is normally only reached during extreme activity. We used this large tidal volume to make clear what results we could obtain from these two respirators. Under more normal circumstances, tidal volumes would be less and so would leakage volumes. How much less is difficult to say, especially for the breathing-demand respirator.

OSHA [7] has published a list of assigned protection factors (APFs) for various classes of respirators. Tight-fitting PAPRs have an assigned protection factor of 1000. Results from this experiment seem to confirm this ruling. Granted that there were small amounts of leakage of air into the facepieces, but amounts were less than the volumes inside the masks, and so not likely to be inhaled, depending on the flow path within the respirator. The lack of fog seen at the mouth indicated that the wearer would have been protected adequately for contaminants acting similar to the fog. Besides, the leakage that occurred, probably as a result of exhalation valve dynamics, was of very short duration and may not result in a significant concentration of contaminant inside the facepiece. 

Protection factors are presently measured by sampling inside and outside the respirator facepiece. The ratio of the two concentrations determines the protection factor. Many of these concentrations are measured with particle counters, but other devices can be used for vapors and gases. These measurements are all site specific and averaged over the sampling time. The position of the sampling tube inside the respirator is usually near the mouth. Unless the contaminant reaches the mouth, little, if any, contaminant is registered, and unless the contaminant is present at the mouth for a long enough time, the average contaminant reading would be small. These are the shortcomings of the present method for determining protection factors. Thus, despite the fact that leakage volumes in this study were measured as 2%–10% of the tidal volume tested, if no contaminant (fog) reached the mouth, the protection factors for these respirators would be found to be extremely high. 

We have investigated leakages in two powered air-purifying respirators from two reputable manufacturers. There is reason to expect that similar types of respirators from other manufacturers would be as safe to wear as these seem to be. In the technological cycle of product improvement, tests such as these can lead to focus on items that limit performance. From such tests come devices that are safer, and, we would hope, more compatible with those who wear them.
